# Supplementary material for: Seasonal and Geographical Impact on the Mycotoxigenicity of Aspergillus and Fusarium Species Isolated from Smallholder Dairy Cattle Feeds and Feedstuffs in Free State and Limpopo Provinces of South Africa
Source: Toxins (Basel). 2023 Feb 4;15(2):128. doi: 10.3390/toxins15020128 (PMC9965880; doi:10.3390/toxins15020128)
Supplement: Supplementary file 1 [file toxins-15-00128-s001.zip › toxins-2161470-supplementary.pdf]

Table S1: Production of AFB_1 and AFB_2 by *A. flavus* isolates in dairy cattle feeds recovered during summer and winter seasons from Free State and Limpopo provinces, SA.

SN	Sample code	Fungal Source	Province	Season	AFB_1 (µg/kg)	AFB_2 (µg/kg)	Total AFs (µg/kg)
1	HS11	TMR	Free State	Winter	14.44	0.26	14.70
2	JF01	Pellet	Limpopo	Summer	1045.8	1.91	1048.71
3	NJ08	Pellet	Limpopo	Summer	84.59	3.44	88.03
4	GF05	Lucerne	Limpopo	Summer	2.16	ND	2.16
5	HS08	TMR	Free State	Summer	576.14	2.42	578.56
6	HS23	Silage	Free State	Winter	0.69	ND	0.69
7	JF06	Pellet	Limpopo	Summer	1.15	0.13	1.28
8	JF08	Pellet	Limpopo	Winter	0.84	ND	0.84
9	GF08	Lucerne	Limpopo	Winter	2.95	0.21	3.16
10	GF03	Grasses	Limpopo	Summer	298.92	0.89	299.81
11	PD01	Soybean	Free State	Summer	0.80	0.11	0.91
12	HS12	Pellet	Free State	Summer	190.22	2.82	193.04
13	GF04	Grasses	Limpopo	Summer	4.64	ND	4.64
14	HS22	TMR	Free State	Winter	1.04	ND	1.04
15	NJ04	Others	Limpopo	Summer	18.85	0.75	19.6
16	HS01	TMR	Free State	Summer	47.34	0.11	47.45
17	PD13	Others	Free State	Winter	3.13	ND	3.13
18	NJ06	Grasses	Limpopo	Summer	2.36	ND	2.36
19	PD03	Others	Free State	Summer	10.88	ND	10.38
20	HS05	Others	Free State	Summer	0.38	ND	0.38
21	NJ02	Pellet	Limpopo	Summer	0.43	ND	0.43
22	HS03	TMR	Free State	Summer	0.38	ND	0.38
23	PD09	TMR	Free State	Summer	0.22	ND	0.22
24	GF06	Lucerne	Limpopo	Summer	0.93	ND	0.93

ND: not detected; AFs: aflatoxins; AFB_1 = aflatoxin B_1; AFB_2: aflatoxin B_2; others: molasses and ramilick; mycotoxins concentrations are recorded in µg/kg.

Table S2. Production of ZEN by *F. oxysporum* and *F. equiseti* isolates in dairy cattle feeds recovered during summer and winter seasons from Limpopo and Free State provinces, SA.

SN	Sample Code	Fungal Source	Fungi Isolate	Province	Season	ZEN (µg/kg)

1	JF05	Pellet	*F. Oxysporum*	Limpopo	Summer	7.80
2	PD11	Lucerne	*F. equiseti*	Free State	Winter	7.64
3	HS02	TMR	*F. Oxysporum*	Free State	Summer	11.09
4	GF06	Pellet	*F. equiseti*	Limpopo	Summer	97.18
5	HS16	Lucerne	*F. Oxysporum*	Free State	Winter	15.90
6	HS02	TMR	*F. equiseti*	Free State	Summer	8.69
7	PD06	Other	*F. Oxysporum*	Free State	Summer	12.52
8	HS03	TMR	*F. Oxysporum*	Free State	Summer	7.75
9	GF08	Lucerne	*F. equiseti*	Limpopo	Winter	9/08
10	HS15	TMR	*F. Oxysporum*	Free State	Winter	5.20
11	JF05	Pellet	*F. equiset*	Limpopo	Summer	19.06
12	GF03	Grasses	*F. Oxysporum*	Limpopo	Summer	16.29

ZEN: zearalenone; others: dairy concentrates; mycotoxins concentrations are recorded in μg/kg.
